# Improving outcome prediction in individuals with colorectal cancer and diabetes by accurate assessment of vascular complications: Implications for clinical practice

**DOI:** 10.1016/j.ejso.2020.10.033

**Published:** 2021-05

**Authors:** Rebecca J. Birch, Amy Downing, Paul J. Finan, Simon Howell, Ramzi A. Ajjan, Eva J.A. Morris

**Affiliations:** a Leeds Institute for Data Analytics, Level 11, Worsley Building, University of Leeds, Leeds, LS2 9JT, UK; b Leeds Institute of Medical Research at St James’s, University of Leeds, Leeds, UK; c Leeds Teaching Hospitals NHS Trust, UK; d Leeds Institute of Cardiovascular and Metabolic Medicine, University of Leeds, Leeds, LS2 9JT, UK; e Nuffield Department of Population Health, Big Data Institute, Old Road Campus, University of Oxford, Headington, Oxford, OX3 7LF, UK

**Keywords:** colorectal cancer, comorbidity, surgery, outcomes, diabetes

## Abstract

**Background:**

Diabetes is considered a risk factor for mortality following a diagnosis of cancer. We hypothesised that the risk will vary due to the heterogeneous nature of the population and accurate classification of vascular complications will improve prediction of clinical outcomes.

**Methods:**

The COloRECTal cancer data Repository (CORECT-R) was used to identify individuals with primary colorectal cancer, who underwent surgical resection in England (2005–2016). Diabetes was recorded using ICD10 codes (E10-E14) during inpatient hospital admission in the six years preceding cancer diagnosis, complication status was determined using the adapted Diabetes Complications Severity Index (aDCSI). Survival and post-operative outcomes were compared between groups.

**Results:**

Of 232,367 individuals, 28,642 (12.3%) were recorded as having diabetes, 49.2% of whom had complications according to the aDCSI. Patients with diabetes complications had increased incidence of adverse post-operative outcomes (90-day post-operative mortality (6.6% versus 3.2%) and death during the surgical episode (7.9% versus 3.6%)), compared to those without diabetes. Those without complications had rates comparable to the population without diabetes. The odds of death within a year of diagnosis were higher for those with complicated diabetes compared to those without diabetes [OR 1.58 (95%CI 1.51–1.66) p < 0.01], but no difference was observed between those with uncomplicated diabetes and those without diabetes [OR 1.05 (95%CI 0.99–1.11) p = 0.10].

**Conclusions:**

Prediction of outcome following surgery in colorectal cancer patients with diabetes relies on the accurate assessment of complications. This study suggests that the poor post-operative outcomes in diabetes patients may be associated with diabetes complication rather than diabetes itself.

## Introduction

Diabetes is prevalent in individuals with cancer, with up to 20% of these patients having disordered glucose metabolism [1]. The presence of diabetes in cancer patients is associated with adverse post-operative outcomes [2] and higher anaesthetic risk [3]. In the context of colorectal cancer, alongside the surgical risk, diabetes has also been associated with lower rates of curative treatment [4], higher rates of chemotherapy related toxicity [5], increased risk of post-operative mortality [6] and lower survival rate [5]. As individuals with diabetes form an increasingly large proportion of the colorectal cancer population, this is of significant concern for clinicians, commissioners, policy makers and, most importantly, patients themselves.

A diagnosis of diabetes has also been shown to be an independent predictor of mortality from colorectal cancer after adjustment for case-mix [7]. However, studies on the role of the complications of diabetes in modulating mortality risk in colorectal cancer patients have been both limited and inconclusive. It is known, however, that complications of diabetes are associated with a lower life expectancy in the general population. This direct relationship between life expectancy and diabetes-related complications, observed amongst the diabetes population, may also exist in the cancer population. In order to fully investigate the relationship between diabetes and cancer outcomes, it is important to accurately differentiate between patients with complicated and uncomplicated diabetes, in order to determine whether diabetes itself is associated with a higher risk or rather the complications stemming from diabetes.

Quality of care measures are applied in cancer population data to identify variations in outcome between different clinical service providers and different geographical areas. In order to do this robustly, case-mix adjustments are used to account for differences between populations including the burden of comorbid disease. In colorectal cancer studies, the Charlson (CCI) [8], Elixhauser (ECI) [9] and C3 [10] comorbidity indices are often used to account for the burden of comorbidity when comparing individual surgeons, treatment centres or National Health Service (NHS) centres in England [11]. Whilst the CCI, ECI and C3 measures include a measure of diabetes severity, they were designed to examine comorbidity as a whole and not solely the impact of diabetes, and were not intended to be used to differentiate between patients with complicated and uncomplicated diabetes. The approach to identifying the complications of diabetes in population data has been refined in recent studies, including a study which has adapted the Diabetes Complications Severity Index (DCSI) for use in population level data, resulting in the aDCSI [12]. However, to date, no population-level studies have attempted to examine the difference in cancer-related outcomes in relation to the complication status of patients with diabetes.

We hypothesised that the risk associated with diabetes is not homogeneous and influenced by the presence of diabetes-related complications. The aim of this work was to identify a reliable and practical system that categorises individuals with diabetes, in order to predict adverse outcome in this population following colorectal surgery. Therefore, we used a large national linked dataset to assess the ability of the aDCSI measure to capture diabetes-related complications and analysed the relationship with post-surgical outcomes.

## Methods

### Study population and data sources

Information was extracted from the COloRECTal cancer data Repository (CORECT-R) for all individuals, diagnosed with a first primary colorectal cancer (ICD10 code C18–C20 [13]) in England between January 1, 2005 and the December 31, 2016, who underwent a major surgical resection of their cancer. Only those who underwent a major surgical resection of their colorectal tumour were included in the analysis. Major surgical resections were identified using the OPCS Classification of Interventions and Procedures (OPCS-4) codes [14] used in Hospital Episode Statistics (HES) data to classify procedures undertaken during a hospital stay. The codes were grouped using previously described methods [15] in order to identify major surgical resection of colorectal cancer.

Information on age at diagnosis, sex, stage of disease, tumour site, route to diagnosis [16], survival time and socioeconomic status (based on the income domain of the Index of Multiple Deprivation (IMD) [17]) was obtained from the National Cancer Registration and Analysis Service (NCRAS) [18] component of this resource. Stage of disease was classified in the cancer registration component of the data (I, II, III, and IV), with an unknown stage group where the data were missing. Details about the surgical management were obtained from the Hospital Episode Statistics (HES) component of CORECT-R [19]. Information about the diagnostic reasons for hospital admissions preceding the diagnosis of the relevant colorectal cancer were also obtained from this source.

Individuals who were identified as having colorectal cancer by Death Certification Only (DCO) [16] were excluded. Due to differences in surgical management and tumour biology, individuals with a tumour of the appendix (C18.1) were also excluded from the analysis.

### Derivation of variables

Emergency surgery was defined as a major surgical resection occurring within 48 h of an emergency inpatient admission, as identified through the admission method reported in the HES data.

#### Diabetes status

Diabetes status was determined from HES data. Individuals were classified as having pre-existing diabetes if the relevant ICD10 codes (E10-E14) had been reported during an inpatient stay within the six years preceding colorectal cancer diagnosis. Six years has been demonstrated previously to be the optimal time period for capturing the presence of comorbidities when using population data [20]. Codes for complications of diabetes were obtained from HES data across the same time period.

The aDCSI measure is a development of the Diabetes Complications Severity Index (DCSI), which is designed to measure diabetes severity in population data. Complication status was determined based on the reporting of ICD10 codes specified for the aDCSI measure (Table A1). Patients were classified into one of three categories; no diabetes, uncomplicated diabetes and complicated diabetes.

**Table 1:** Characteristics of the surgical study population by aDCSI status.

		No diabetes		Uncomplicated diabetes		Complicated diabetes		p value
		N	%	n	%	N	%	
Age	<40	3526	1.7	57	0.4	17	0.1	<0.01
	40–49	8739	4.3	319	2.2	95	0.7	
	50–59	26,152	12.8	1463	10.1	629	4.5	
	60–69	57,982	28.5	4439	30.5	2979	21.1	
	70–79	66,219	32.5	5711	39.2	6229	44.2	
	≥80	41,108	20.2	2562	17.6	4141	29.4	
Median age (IQR)		70 (62–78)		71 (64–77)		75 (68–81)		<0.01
Sex	Male	112,558	55.2	8685	59.7	9445	67.0	<0.01
	Female	91,168	44.8	5866	40.3	4645	33.0	
Socioeconomic status	1 - most affluent	45,705	22.4	2645	18.2	2361	16.8	<0.01
	2	47,663	23.4	3094	21.3	2765	19.6	
	3	42,668	20.9	3076	21.1	2922	20.7	
	4	36,806	18.1	3025	20.8	3028	21.5	
	5 - most deprived	30,884	15.2	2712	18.6	3014	21.4	
Tumour site	Colon	135,659	66.6	10,310	70.9	10,747	76.3	<0.01
	Rectosigmoid	14,145	6.9	938	6.4	814	5.8	
	Rectum	53,922	26.5	3303	22.7	2529	17.9	
Stage of disease	I	29,438	14.4	2118	14.6	2054	14.6	<0.01
	II	68,504	33.6	5007	34.4	5172	36.7	
	III	70,367	34.5	5145	35.4	4780	33.9	
	IV	17,660	8.7	1238	8.5	1078	7.7	
	Unknown	17,757	8.7	1043	7.2	1006	7.1	
Admission method	Elective	165,637	81.3	11,863	81.5	10,885	77.3	<0.01
	Emergency	38,039	18.7	2685	18.5	3203	22.7	
	Unknown	50	0.0	3	0.0	2	0.0	
		203,725		14,551		14,090		

#### Surgical outcomes

Outcomes studied were selected based on their use as standardised endpoints and quality of care indicators in post-operative medicine and colorectal cancer surgery [11,21].

Three month post-operative mortality was defined as all-cause mortality within 90-days of the definitive major surgical resection for colorectal cancer, calculated using the date of surgery and date of death [11,21]. As in the National Bowel Cancer Audit, the observed rates of 90-day post-operative mortality were calculated for elective and emergency surgery separately [11].

Death within the surgical episode was defined as a death occurring within the same admission to an NHS hospital as the major surgical resection of the colorectal tumour. It represents the proportion of patients who were never discharged from hospital following their major surgical resection.

Analysis of readmission rates and prolonged length of stay included only patients who had survived the surgical episode. Readmission was defined as an admission to hospital within 30 days of the surgical inpatient spell [11,21]. Length of stay was calculated from the time (in days) between the major surgical resection and discharge from hospital [21], prolonged length of stay was determined as being a length of inpatient hospital stay of 21 or more days following major surgical resection [22].

#### Survival

Survival was calculated, in years, from the date of diagnosis to the date of death or censoring (December 31, 2016). One-year mortality was produced as a binary variable. Individuals were classified as having died within one year of their colorectal cancer diagnosis if their survival was less than 365 days. The impact of components of the aDCSI score was assessed against one-year mortality in order to assess the relationship between complications of diabetes and outcomes from cancer. Complications are known to influence life expectancy and so a longer time frame is likely to capture this effect, rather than the relationship with cancer.

### Statistical analysis

Descriptive analyses were performed to assess the variation in outcomes in relation to the presence or absence of diabetes and its complications. *χ*^2^ analysis was performed to test for significant differences in the characteristics between the three groups (no diabetes, complicated diabetes and uncomplicated diabetes). A nonparametric test of medians was undertaken to compare the age at diagnosis across the groups. Kaplan-Meier survival estimates were calculated with the survival function calculated at five years.

In order to assess the relationship between diabetes complication status (using the aDCSI measure) and one-year mortality, adjusted logistic regression was used to calculate the odds of death within one year of CRC diagnosis for those who had undergone a major surgical resection. The model included all individuals who had undergone resection, with adjustment for, age at CRC diagnosis, sex, socioeconomic status, stage of disease, tumour site and year of CRC diagnosis. Missing data were included as a separate category within each variable. To assess the relationship between each component of the aDCSI measure and death within one year of diagnosis, a second logistic regression model was developed which included only patients with diabetes-related complications and looked at the diagnosis of retinopathy, nephropathy, neuropathy, cerebrovascular disease, cardiovascular disease and peripheral vascular disease. These were included in the same model as multiple complications may be present in each individual. This model was adjusted for the same characteristics as the first model.

All statistical analyses were undertaken in Stata 15.0.

## Results

Within the study population of 232,367 individuals with colorectal cancer diagnosed between January 1, 2005 and December 31, 2016 who underwent a major surgical resection of their colorectal tumour, 28,641 (12.3%) were recorded as having diabetes.

### Patient and tumour characteristics and diabetes status

Overall, 49.2% (n = 14,090) of those with diabetes who underwent a major surgical resection of their colorectal cancer were considered to have one or more diabetes complications.

Patients classified as having complicated diabetes were older than those classified as having no diabetes or uncomplicated diabetes, median (IQR) age at CRC diagnosis 75 (68–81) years versus 70 (62–78) and 71 (64–77) years respectively (p < 0.01) (Table 1). A higher proportion of those who in the complicated group were male patients (67.0% (n = 9445) compared to individuals without diabetes and those with uncomplicated diabetes (55.2% (n = 112,558) and 59.7% (n = 8685) respectively) (p < 0.01). Individuals with complicated diabetes also had a higher proportion of colon tumours (76.3%, n = 10,747), emergency admission for major surgical resection (22.7%, n = 3203) and lived in areas with higher levels of socioeconomic deprivation (21.4%, n = 3014) than those without diabetes (66.6%, 18.7% and 15.2% respectively) and those with uncomplicated diabetes (70.9%, 18.5% and 18.6% respectively) (p < 0.01) (Table 1).

### Post-operative outcomes and diabetes status

**Fig. 1:**
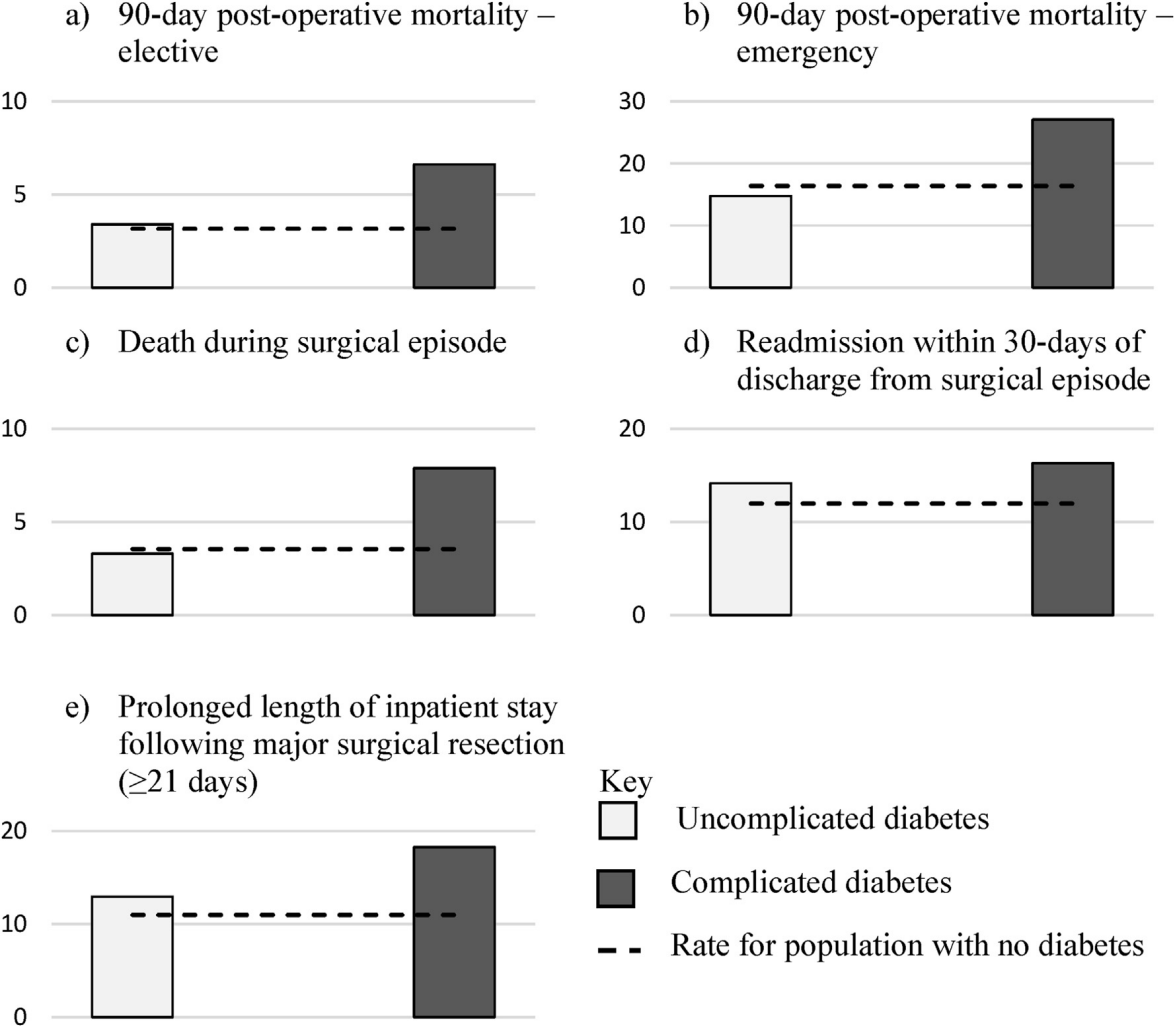
Outcomes from colorectal cancer by diabetes complications status.

Patients identified as having diabetes complications suffered an increased burden of adverse outcomes when compared to those without diabetes. These included an increased rate of 90-day post-operative mortality (for both elective and emergency surgery), death during the surgical episode, readmission and prolonged length of stay (Fig. 1). In contrast, the rate of post-operative mortality and surgical deaths amongst individuals with no complications was comparable to that of the population without diabetes. (Fig. 1).

### Survival and diabetes status

**Fig. 2:**
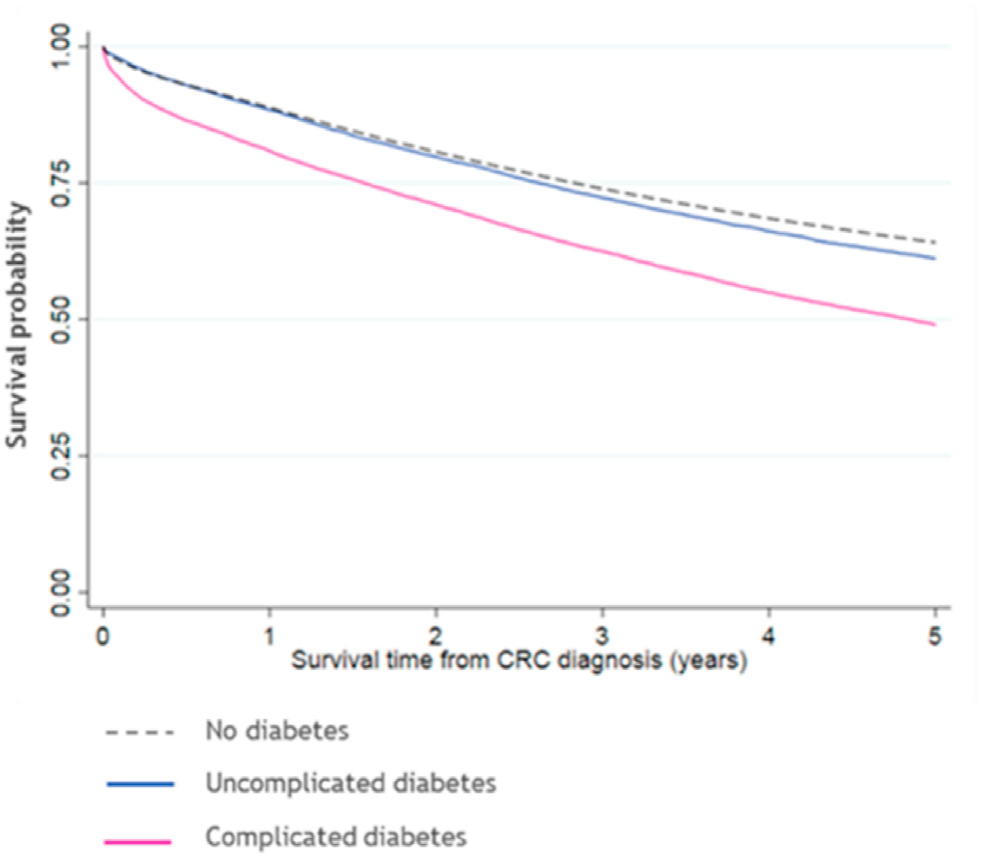
Kaplan-Meier survival estimates for those who underwent a major surgical resection of their colorectal cancer, by diabetes complications status (number at risk at each time point and survivor function available in Table A2).

The longer-term survival of patients classified as having complicated diabetes was significantly worse than the population without diabetes (Fig. 2). The five-year survival function (SF) of those classified as having uncomplicated diabetes [SF 0.61 (95%CI 0.60–0.62)] was comparable to that of individuals without diabetes [SF 0.64 (95%CI 0.64–0.64)].

**Fig. 3:**
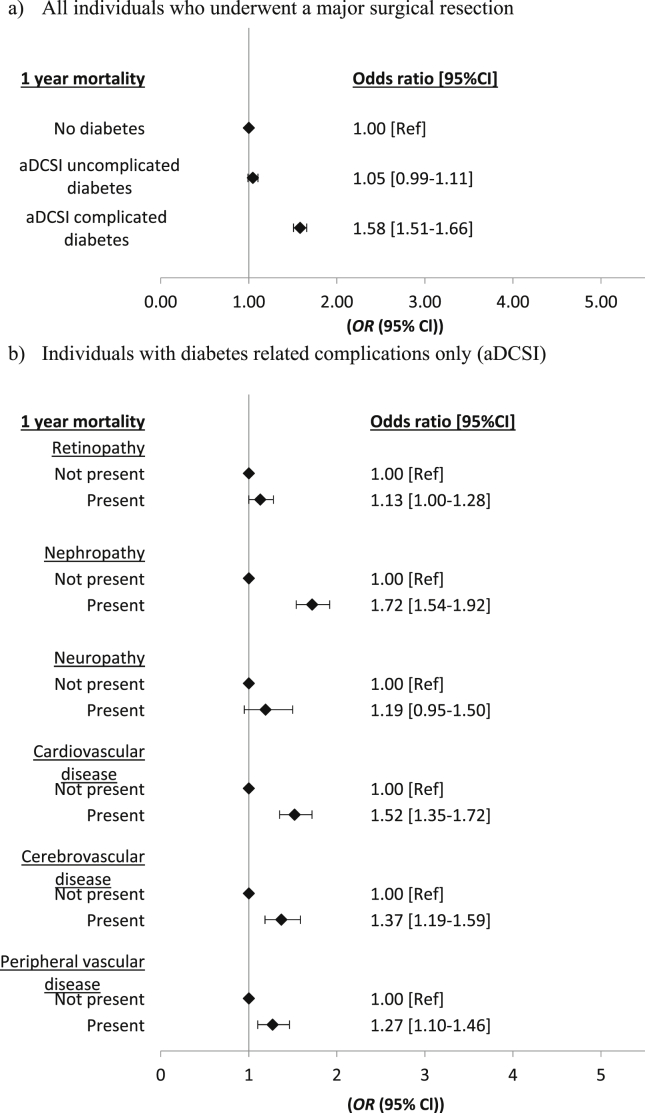
Odds of death within a year of colorectal cancer diagnosis according to diabetes complication status and type of complication. Models adjusted for age, sex, socioeconomic status, stage of CRC, site of tumour and year of CRC diagnosis (full results of adjusted models available in Table A3 & Table A4.).

Having complicated diabetes was associated with increased odds of death within one year of diagnosis compared to those with either no diabetes or uncomplicated diabetes [OR 1.58 (95%CI 1.51–1.66); p < 0.01) (Fig. 3a). The different components of the aDCSI complication score were associated with different one-year survival estimates. Patients with nephropathy had significantly higher odds of death within 1 year [OR 1.72 (95%CI 1.54–1.92; p < 0.01] when compared to those without (Fig.4b). Cardiovascular disease, cerebrovascular disease and peripheral vascular disease were also all associated with a significantly increased risk of one-year mortality (Fig. 3b). The odds of death in those with retinopathy showed an increase, although this only showed a trend (OR 1.13 95%CI 1.0–1.28 p = 0.05) while neuropathy failed to show an association [OR 1.19 (95%CI 0.95–1.50); p = 0.13].

## Discussion

This is the first study to seek to quantify the impact of diabetes-related complications in a cancer population who have undergone major surgery. Our data suggests that the adverse outcome in diabetes patients undergoing surgery for colorectal cancer is related to complications of the condition, rather than diabetes itself. Moreover, this study demonstrates that using the aDCSI for identification of diabetes complications in epidemiological data increases the ability to predict clinical outcome.

This study shows that the burden of adverse post-operative outcomes in colorectal cancer patients with diabetes appears to rest with those who have one or more of the complications of diabetes. Conversely, patients who do not have complications of diabetes had a similar prognosis to those with normal glucose metabolism.

Both the diabetes and complication status of individuals being derived from secondary care data, which could lead to an under capture of individuals managed solely by primary care. In our study, the proportion of individuals with diabetes and those who were identified as having diabetes-related complications using the aDCSI measure generally reflects what has been observed in the diabetes population [23]. This suggests that the ascertainment of cases is not significantly flawed by this approach.

These data have implications for risk stratification and management of CRC patients. The aDCSI measure provides the opportunity to assess the burden of diabetes complications at a population level. Evidence-based counselling and clinical management of colorectal cancer patients with diabetes requires knowledge of their burden of diabetes complications. Effective risk adjustment in epidemiological studies and national cancer audits further requires the accurate ascertainment of diabetic complications and the aDCSI appears to achieve this.

The exact mechanisms for the adverse clinical outcome in individuals with diabetes and the associated vascular complications is not always clear but likely to be multifactorial. Individuals with diabetes are more prone to post-operative complications which may have both a short and a long term impact: gut motility is believed to be slower in returning after surgery in those with diabetes [24], surgical site infection occurs more frequently in this group [25] and these individuals are more prone to anastomotic leak [26]. Whilst these complications can be devastating, our data suggest that at population level, the cardiovascular, cerebrovascular and renal complications of diabetes are key predictive factors of adverse clinical outcome in colorectal cancer patients with diabetes.

Observational data indicate that patients with diabetes who develop cancer receive less aggressive treatment [27]. The reasons for this are not fully understood and are likely to be complex. A perception that all individuals with diabetes are at greater risk of adverse outcome may play a part in treatment decisions. However, our data demonstrate that postoperative outcomes in colorectal cancer patients with uncomplicated diabetes are broadly comparable to the population without diabetes and should therefore be managed accordingly. Further work to examine this in the context of adjuvant treatment is needed. Whilst the impact of adjuvant treatment on the immediate post-operative outcomes is likely to be minimal, variation in treatment rates amongst the study groups may influence the longer term outcomes.

There is an extensive literature on the association between HbA1c, an indicator of average glycaemic control, and post-operative outcome. While HbA1c was not included in the measure of diabetes severity in the current work, aDCSI has been tested against a measure which includes HbA1c and omission of this glycaemic marker did not adversely affect the predictive ability of the score [12].

Appropriate risk adjustment for the impact of comorbidity on outcome is important not only for epidemiological studies but for the audit and governance of cancer services and clinical practice. The advent of public reporting of hospital and surgical performance supports transparency and quality improvement. However, it brings with it a duty to provide the highest possible quality data with effective statistical adjustment for case mix and comorbidities.

In conclusion, this study demonstrates that the risk associated with diabetes in patients undergoing surgery for colorectal cancer is related to vascular complications of the condition, rather than diabetes per se. Both renal and macrovascular complications (cardiovascular, peripheral vascular and cerebrovascular) are strongly associated with postoperative complications and death following major surgery. The optimal management of patients with these conditions is at the core of research and developments in post-operative care [28,29]. Preoperative risk stratification, to allow targeted care, is at the core of the management of the high-risk surgical patient. These results have potential impact for policy makers, clinicians and patients, and demonstrate the importance of adjustment for diabetes status in order to avoid inappropriate inflation of the risk associated with uncomplicated diabetes. Appropriate classification of individuals with diabetes and colorectal cancer will help the health care professional in improving prediction of outcome and adjusting management accordingly.

## CRediT authorship contribution statement

**Rebecca J. Birch:** Conceptualization, Methodology, Validation, Formal analysis, Writing - original draft, Writing - review & editing. **Amy Downing:** Methodology, Validation, Formal analysis, Writing - original draft, Writing - review & editing. **Paul J. Finan:** Writing - review & editing. **Simon Howell:** Conceptualization, Validation, Writing - original draft, Writing - review & editing. **Ramzi A. Ajjan:** Conceptualization, Validation, Writing - original draft, Writing - review & editing. **Eva J.A. Morris:** Validation, Writing - review & editing, Funding acquisition.

## Data Availability

The data used in this study are available from the National Cancer Registration and Analysis Service via the Public Health England Office for Data Release and the UK Colorectal Cancer Intelligence Hub, subject to relevant approvals.
