# Supplementary material for: Improving outcome prediction in individuals with colorectal cancer and diabetes by accurate assessment of vascular complications: Implications for clinical practice
Source: Eur J Surg Oncol. 2021 May;47(5):999–1004. doi: 10.1016/j.ejso.2020.10.033 (PMC8117971; doi:10.1016/j.ejso.2020.10.033)
Supplement: Multimedia component 1 [file mmc1.docx]

**SUPPLEMENTARY TABLES**

Appendix table 1: Adapted Diabetes Complications Severity Index

| ICD10 code | Description | Uncomplicated | Complicated | aDCSI points | Complication category |
| --- | --- | --- | --- | --- | --- |
| E109 | Insulin-dependent diabetes mellitus without complication |  |  |  |  |
| E119 | Non-insulin-dependent diabetes mellitus without complications |  |  |  |  |
| E129 | Malnutrition-related diabetes mellitus without complications |  |  |  |  |
| E139 | Other specified diabetes mellitus without complications |  |  |  |  |
| E149 | Unspecified diabetes mellitus without complications |  |  |  |  |
| E103 | Insulin-dependent diabetes mellitus with ophthalmic complications |  |  | 1 | Ocular |
| E113 | Non-insulin-dependent diabetes mellitus with ophthalmic complications |  |  | 1 |  |
| E123 | Malnutrition-related diabetes mellitus with ophthalmic complications |  |  | 1 |  |
| E133 | Other specified diabetes mellitus with ophthalmic complications |  |  |  |  |
| E143 | Unspecified diabetes mellitus with ophthalmic complications |  |  | 1 |  |
| H280 | Diabetic cataract |  |  | 1 |  |
| H350 | Background retinopathy and retinal vascular changes |  |  | 1 |  |
| H353 | Degeneration of macula and posterior pole |  |  | 1 |  |
| H356 | Retinal haemorrhage |  |  | 1 |  |
| H358 | Other specified retinal disorders |  |  | 1 |  |
| H360 | Diabetic retinopathy |  |  | 1 |  |
| H352 | Other proliferative retinopathy |  |  | 2 |  |
| H330 | Retinal detachment and breaks |  |  | 2 |  |
| H540 | Blindness, binocular |  |  | 2 |  |
| H544 | Blindness, monocular |  |  | 2 |  |
| H431 | Vitreous haemorrhage |  |  | 2 |  |
| E102 | Insulin-dependent diabetes mellitus with renal complications |  |  | 1 | Renal |
| E112 | Non-insulin-dependent diabetes mellitus with renal complications |  |  | 1 |  |
| E122 | Malnutrition-related diabetes mellitus with renal complications |  |  |  |  |
| E132 | Other specified diabetes mellitus with renal complications |  |  | 1 |  |
| E142 | Unspecified diabetes mellitus with renal complications |  |  | 1 |  |
| N083 | Glomerular disorders in diabetes mellitus |  |  | 1 |  |
| N03 | Chronic nephritic syndrome |  |  | 1 |  |
| N04 | Nephrotic syndrome |  |  | 1 |  |
| N05 | Unspecified nephritic syndrome |  |  | 1 |  |
| N17 | Acute renal failure |  |  | 2 |  |
| N18 | Chronic kidney disease |  |  | 2 |  |
| N19 | Unspecified kidney failure |  |  | 2 |  |
| T824 | Mechanical complication of vascular dialysis catheter |  |  | 2 |  |
| Z49 | Care involving dialysis |  |  | 2 |  |
| Z992 | Dependence on renal dialysis |  |  | 2 |  |
| E104 | Insulin-dependent diabetes mellitus with neurological complications |  |  | 1 | Neurological |
| E114 | Non-insulin-dependent diabetes mellitus with neurological complications |  |  | 1 |  |
| E124 | Malnutrition-related diabetes mellitus with neurological complications |  |  | 1 |  |
| E134 | Other specified diabetes mellitus with neurological complications |  |  | 1 |  |
| E144 | Unspecified diabetes mellitus with neurological complications |  |  | 1 |  |
| G609 | Hereditary and idiopathic neuropathy, unspecified |  |  | 1 |  |
| G730 | Myasthenic syndromes in endocrine diseases |  |  | 1 |  |
| H490 | Third (oculomotor) nerve palsy |  |  | 1 |  |
| H491 | Fourth (trochlear) nerve palsy |  |  | 1 |  |
| H492 | Sixth (abducent) nerve palsy |  |  | 1 |  |
| G590 | Diabetic mononeuropathy |  |  | 1 |  |
| M146 | Neuropathic arthropathy |  |  | 1 |  |
| M142 | Diabetic arthropathy |  |  | 1 |  |
| G632 | Diabetic polyneuropathy |  |  | 1 |  |
| G990 | Autonomic neuropathy in endocrine and metabolic disorders |  |  | 1 |  |
| G45 | Transient cerebral ischaemic attacks and related syndromes |  |  | 1 | Cerebrovascular |
| I61 | Intracerebral haemorrhage |  |  | 1 |  |
| I63 | Cerebral infarction |  |  | 1 |  |
| I64 | Stroke, not specified as haemorrhage or infarction |  |  | 1 |  |
| I69 | Sequelae of cerebrovascular disease |  |  | 1 |  |
| I70 | Atherosclerosis |  |  | 1 | Cardiovascular |
| I24 | Other acute ischemic heart diseases |  |  | 1 |  |
| I20 | Angina pectoris |  |  | 1 |  |
| I25 | Chronic ischemic heart disease |  |  | 1 |  |
| I21 | Acute myocardial infarction |  |  | 2 |  |
| I23 | Certain current complications following acute myocardial infarction |  |  | 2 |  |
| I490 | Ventricular fibrillation and flutter |  |  | 2 |  |
| I46 | Cardiac arrest |  |  | 2 |  |
| I48 | Atrial fibrillation and flutter |  |  | 2 |  |
| I22 | Subsequent myocardial infarction |  |  | 2 |  |
| I50 | Heart failure |  |  | 2 |  |
| I110 | Hypertensive heart disease with (congestive) heart failure |  |  | 2 |  |
| I130 | Hypertensive heart and renal disease with (congestive) heart failure |  |  | 2 |  |
| I71 | Aortic aneurysm and dissection |  |  | 2 |  |
| E105 | Insulin-dependent diabetes mellitus with peripheral circulatory complications |  |  | 1 | Peripheral vascular |
| E115 | Non-insulin-dependent diabetes mellitus with peripheral circulatory complications |  |  | 1 |  |
| E125 | Malnutrition-related diabetes mellitus with peripheral circulatory complications |  |  | 1 |  |
| E135 | Other specified diabetes mellitus with peripheral circulatory complications |  |  | 1 |  |
| E145 | Unspecified diabetes mellitus with peripheral circulatory complications |  |  | 1 |  |
| I792 | Peripheral angiopathy in diseases classified elsewhere |  |  | 1 |  |
| I724 | Aneurysm and dissection of artery of lower extremity |  |  | 1 |  |
| I739 | Peripheral vascular disease, unspecified |  |  | 1 |  |
| I743 | Embolism and thrombosis of arteries of lower extremities |  |  | 2 |  |
| R02 | Gangrene, not elsewhere classified |  |  | 2 |  |
| I702 | Atherosclerosis of arteries of extremities |  |  | 2 |  |
| A480 | Gas gangrene |  |  | 2 |  |
| L97 | Non pressure chronic ulcer of lower limb, not elsewhere classified |  |  | 2 |  |
| L984 | Non pressure chronic ulcer of skin, not elsewhere classified |  |  | 2 |  |

Appendix table 2: Number at risk at each time point and survivor functions calculated during survival analysis

|  | Time from CRC diagnosis | | | | | |  | Survival function at 5 years | |
| --- | --- | --- | --- | --- | --- | --- | --- | --- | --- |
|  | 0 | 1 | 2 | 3 | 4 | 5 |  | Survivor function | 95%CI |
| No diabetes | 203,726 | 180,438 | 150,494 | 124,938 | 103,751 | 85,763 |  | 0.64 | (0.64-0.64) |
| Uncomplicated diabetes | 14,551 | 12,810 | 10,300 | 8,204 | 6,504 | 5,100 |  | 0.61 | (0.60-0.62) |
| Complicated diabetes | 14,090 | 11,350 | 8,919 | 6,856 | 5,209 | 3,929 |  | 0.49 | (0.48-0.50) |

Appendix table 3: Adjusted logistic regression model for 1-year mortality, results for all individuals who underwent a major surgical resection of their colorectal cancer

|  |  | OR | P value | Lower 95%CI | Upper 95%CI |
| --- | --- | --- | --- | --- | --- |
| aDCSI diabetes status | No diabetes | 1.00 |  |  |  |
|  | Uncomplicated diabetes | 1.05 | 0.10 | 0.99 | 1.11 |
|  | Complicated diabetes | 1.58 | <0.01 | 1.51 | 1.66 |
| Age at CRC diagnosis (single year increase) | | 1.06 | <0.01 | 1.05 | 1.06 |
| Sex | Male | 1.00 |  |  |  |
|  | Female | 0.95 | <0.01 | 0.93 | 0.98 |
| Socioeconomic status | 1 – most affluent | 1.00 |  |  |  |
|  | 2 | 1.10 | <0.01 | 1.06 | 1.15 |
|  | 3 | 1.17 | <0.01 | 1.12 | 1.22 |
|  | 4 | 1.31 | <0.01 | 1.26 | 1.37 |
|  | 5 – most deprived | 1.54 | <0.01 | 1.47 | 1.60 |
| Stage of disease | I |  |  |  |  |
|  | II | 1.63 | <0.01 | 1.53 | 1.73 |
|  | III | 3.21 | <0.01 | 3.03 | 3.40 |
|  | IV | 11.22 | <0.01 | 10.54 | 11.95 |
|  | Unknown | 3.65 | <0.01 | 3.41 | 3.91 |
| Site of tumour | Colon |  |  |  |  |
|  | Rectosigmoid | 0.70 | <0.01 | 0.66 | 0.74 |
|  | Rectum | 0.54 | <0.01 | 0.52 | 0.56 |
| Year of CRC diagnosis (single year increase) | | 0.93 | <0.01 | 0.93 | 0.94 |

Appendix table 4: Adjusted logistic regression model for 1-year mortality, results for individuals with complicated diabetes who underwent a major surgical resection of their colorectal cancer

|  |  | OR | P value | Lower 95%CI | Upper 95%CI |
| --- | --- | --- | --- | --- | --- |
| Retinopathy | Not present | 1.00 |  |  |  |
|  | Present | 1.13 | 0.05 | 1.00 | 1.28 |
| Nephropathy | Not present | 1.00 |  |  |  |
|  | Present | 1.72 | <0.01 | 1.54 | 1.92 |
| Neuropathy | Not present | 1.00 |  |  |  |
|  | Present | 1.19 | 0.13 | 0.95 | 1.50 |
| Cardiovascular disease | Not present | 1.00 |  |  |  |
|  | Present | 1.52 | <0.01 | 1.35 | 1.72 |
| Cerebrovascular disease | Not present | 1.00 |  |  |  |
|  | Present | 1.37 | <0.01 | 1.19 | 1.59 |
| Peripheral vascular disease | Not present | 1.00 |  |  |  |
|  | Present | 1.27 | <0.01 | 1.10 | 1.46 |
| Age at CRC diagnosis (single year increase) | | 1.05 | <0.01 | 1.04 | 1.06 |
| Sex | Male | 1.00 |  |  |  |
|  | Female | 0.96 | 0.46 | 0.88 | 1.06 |
| Socioeconomic status | 1 – most affluent | 1.00 |  |  |  |
|  | 2 | 1.09 | 0.24 | 0.94 | 1.27 |
|  | 3 | 1.17 | 0.03 | 1.01 | 1.36 |
|  | 4 | 1.21 | 0.01 | 1.05 | 1.41 |
|  | 5 – most deprived | 1.30 | <0.01 | 1.12 | 1.50 |
| Stage of disease | I | 1.00 |  |  |  |
|  | II | 1.54 | <0.01 | 1.29 | 1.83 |
|  | III | 2.61 | <0.01 | 2.20 | 3.10 |
|  | IV | 9.03 | <0.01 | 7.38 | 11.05 |
|  | Unknown | 3.09 | <0.01 | 2.49 | 3.82 |
| Tumour site | Colon | 1.00 |  |  |  |
|  | Rectosigmoid | 0.82 | 0.05 | 0.67 | 1.00 |
|  | Rectum | 0.72 | <0.01 | 0.63 | 0.82 |
| Year of CRC diagnosis (single year increase) | | 0.92 | <0.01 | 0.91 | 0.93 |
